# Editorial: The affective aspects of chronic pain and potential treatments

**DOI:** 10.3389/fnbeh.2023.1209561

**Published:** 2023-05-10

**Authors:** Dan Kaufmann, Brandon C. Yarns, Parisa Gazerani

**Affiliations:** ^1^Department of Neurology, University of Utah, Salt Lake City, UT, United States; ^2^Department of Mental Health/Psychiatry, VA Greater Los Angeles Healthcare System, Los Angeles, CA, United States; ^3^Department of Psychiatry and Biobehavioral Sciences, University of California, Los Angeles, Los Angeles, CA, United States; ^4^Faculy of Health Sciences, Department of Life Sciences and Health, Oslo Metropolitan University, Oslo, Norway; ^5^Faculty of Medicine, Department of Health Science and Technology, Aalborg University, Aalborg, Denmark

**Keywords:** emotion, biopsychosocial, affect, pain treatment, pain, chronic pain

Chronic pain is a major cause of disability and disease burden globally. Various forms of chronic pain diminish the quality of life of affected individuals, their families, and society (Mills et al., 2019). The pathophysiology of chronic pain has been targeted by various medical interventions, including pharmacological and non-pharmacological strategies (Gatchel et al., 2014; Jensen and Turk, 2014; Edwards et al., 2016). However, these interventions seem often to remain insufficient for treating chronic pain (Jensen and Turk, 2014). An increasing body of evidence indicates a bi-directional relationship between psychosocial factors and chronic pain (Kroenke et al., 2011; Goesling et al., 2018). It has been reported that in half of the cases of depression, a physical pain symptom is present (Katona et al., 2005). Moreover, chronic pain increases the risk of psychological and mood disorders (Bair et al., 2003; Asmundson and Katz, 2009; Edwards et al., 2011; IsHak et al., 2018). This complex co-occurrence has been represented by the biopsychosocial model to emphasize the interaction between physiological, psychological, and social factors in understanding and treating chronic pain (Gatchel et al., 2014). The identification of specific mechanisms underlying interactions between the components of this model has attracted significant attention, for instance, how psychological and social factors influence pain pathways in the brain (Edwards et al., 2016).

This Research Topic (The affective aspects of chronic pain and potential treatments) focused on the affect dimension of chronic pain and aimed to collect novel research to further expand our understanding of the relationship between affect and chronic pain. Five research studies in this collection provide further evidence on the following topics: 1. The changes in brain regions responsible for cognitive/affective tasks in chronic pain patients compared to controls; 2. The importance of social attitudes/beliefs about pain to patient's coping strategies; 3. Preclinical rodent research evaluating the effects of pain models on affect-related behavioral outputs. We believe these important studies contribute to advancing our understanding of the multidimensional nature of pain. This collection also encourages further research to characterize the mechanisms underlying pain and consequently how to target them.

In their paper, Goldway et al. measured steady-state visually evoked potentials in response to affective distractors during a cognitive attention task in fibromyalgia patients compared to controls. Visual attentional biases have previously been linked with the course and severity of several affective disorders (Armstrong and Olatunji, 2012), and using similar methodologies, attention processes have been evaluated in pain-related disorders (Chan et al., 2020). Several other studies have indicated that chronic pain patients allocate attention and process information differently than their pain-free counterparts by fixating more on pain-related stimuli than neutral stimuli (Chan et al., 2020). Moreover, attentional bias may explain the links between cognition, affect, and pain; however, the mechanism is still not fully understood (Pincus and Morley, 2001). In their study, Goldway et al. showed that patients with fibromyalgia had sustained attention to negative cues and impaired affective discrimination during cognitive tasks, compared to controls, which also correlated with pain severity. They also showed that fibromyalgia patients had decreased fronto-occipital task connectivity, which correlated with poor sleep. This study raises the hypothesis of evaluating cognitive-affective attention bias as a measure of disease chronification or a means to evaluate treatment efficacy and therefore opens the door to future longitudinal studies. Moreover, whether these brain changes which are seen in fibromyalgia can be seen in other chronic pain conditions is still unknown.

Another study, by Dobos et al. used fMRI in people living with migraine and controls in order to evaluate brain changes in response to an implicit face emotion processing task. The authors found differences between the groups in brain activity in the supplemental motor area (SMA) and in the insula (the hub of affective processing) in response to fearful and happy faces, respectively. They also evaluated brain changes in both groups in response to a 16-week Autogenic Training (AT), which is considered an evidence-based self-regulation and stress reduction method. While migraine frequency was reduced as a result of AT, the authors found that AT also reduced activation in the parabrachial complex in migraineurs, as well as reduced activation of the SMA and increased activation in the insula in response to happy faces. These changes in the migraine group produced by AT suggested normalization of affect processing and increased openness to positive affect. It is well-known that people living with migraine also experience other psychiatric comorbidities such as anxiety and depression, as well as altered processing in psychological domains such as mood (Marino et al., 2010), tiredness (Raggi et al., 2012), and unpleasantness of environmental stimuli (Demarquay et al., 2006). Yet the mechanism of abnormal emotional processing in migraine is still not completely understood. The changes in activity in the emotional processing areas seen after AT in migraine as reported by Dobos et al. further confirm the importance of these areas in migraine pathophysiology.

The biopsychosocial model of pain asserts the importance of psychosocial influence on chronic pain. Several psychosocial factors can contribute to the development, long-term consequences, and treatment outcomes in chronic pain patients (Edwards et al., 2016). In their study, Alinajimi et al. evaluated the relationship between family caregivers' pain-related attitudes and beliefs and the coping strategies used by chronic musculoskeletal pain patients. They found that the emotion regulation of both patients and caregivers mediates this relationship; therefore, providing emotion regulation strategies is an important consideration when treating chronic pain. Overall, this study identified an important social and interpersonal factor and its influence on pain-related outcomes and highlighted the importance of educating chronic pain patients and their caregivers on emotion regulation and its effects on pain coping.

This Research Topic also included two pre-clinical studies which evaluated behavioral alteration as a result of painful procedures in rodent models. Ririe et al. evaluated the effects of early-life surgery and anesthesia on long-term anxiety, depression, audiovisual attention, and opioid reward behaviors. They found that the occurrence of a painful procedure early in life caused maladaptive behaviors, including increased anxiety, reduced premature responses in an attention task, and greater escalation of heroin intake, which were evaluated long after the procedure was done. Similarly, Nunez-Badinez et al. evaluated the presence of anxiety behaviors in a mouse model of endometriosis. Using the elevated plus maze and the novel environment-induced feeding suppression models, the authors confirmed the presence of anxiety-related behaviors in endometriosis-induced mice. The results of this study provide further evidence of a link between anxiety and endometriosis, which are often comorbid in patients (Facchin et al., 2017; Laganà et al., 2017). Together, these studies further establish the relationship between painful procedures and changes in affective and behavioral measures in pre-clinical models. The specific mechanism which can explain the affective and behavioral changes resulting from painful procedures is not entirely known; therefore, these studies open the door for future mechanistic evaluation of these reported phenotypes.

Overall, this Research Topic provided additional important evidence for the complex relationships between affect and different chronic pain conditions and opens new avenues for future studies to understand specific mechanisms underlying this complex relationship. We would like to thank the authors for their important contributions to this special collection. We also appreciate the support we received from Frontiers for their editorial contributions throughout the process. We hope that this collection will contribute to expanding research in this fascinating field and to the development of novel treatment options for chronic pain.

## Author contributions

All authors listed have made a substantial, direct, and intellectual contribution to the work and approved it for publication.
